# Face Spoofing, Age, Gender and Facial Expression Recognition Using Advance Neural Network Architecture-Based Biometric System

**DOI:** 10.3390/s22145160

**Published:** 2022-07-09

**Authors:** Sandeep Kumar, Shilpa Rani, Arpit Jain, Chaman Verma, Maria Simona Raboaca, Zoltán Illés, Bogdan Constantin Neagu

**Affiliations:** 1 Department of Computer Science and Engineering, Koneru Lakshmaiah Education Foundation, Vaddeswaram, Vijaywada 522302, India; 2 Department of IT, Neil Gogte Institute of Technology, Kachawanisingaram Village, Hyderabad 500039, India; shilpachoudhary1987@gmail.com; 3 College of Computing Sciences and IT, Teerthanker Mahaveer University, Moradabad 244001, India; arpit.computers@tmu.ac.in; 4 Department of Media and Educational Informatics, Faculty of Informatics, Eötvös Loránd University, 1053 Budapest, Hungary; chaman@inf.elte.hu (C.V.); illes@inf.elte.hu (Z.I.); 5 National Research and Development Institute for Cryogenic and Isotopic Technologies—ICSI Rm, 240050 Ramnicu Valcea, Romania; 6 Doctoral School, Polytechnic University of Bucharest, 313 Splaiul Independentei, 060042 Bucharest, Romania; 7 Department of Power Engineering, “Gheorghe Asachi” Technical University of Iasi, 700050 Iasi, Romania; bogdan.neagu@tuiasi.ro

**Keywords:** face detection, U-Net, Alex-Net, facial expression, face spoofing, age, gender

## Abstract

Nowadays, the demand for soft-biometric-based devices is increasing rapidly because of the huge use of electronics items such as mobiles, laptops and electronic gadgets in daily life. Recently, the healthcare department also emerged with soft-biometric technology, i.e., face biometrics, because the entire data, i.e., (gender, age, face expression and spoofing) of patients, doctors and other staff in hospitals is managed and forwarded through digital systems to reduce paperwork. This concept makes the relation friendlier between the patient and doctors and makes access to medical reports and treatments easier, anywhere and at any moment of life. In this paper, we proposed a new soft-biometric-based methodology for a secure biometric system because medical information plays an essential role in our life. In the proposed model, 5-layer U-Net-based architecture is used for face detection and Alex-Net-based architecture is used for classification of facial information i.e., age, gender, facial expression and face spoofing, etc. The proposed model outperforms the other state of art methodologies. The proposed methodology is evaluated and verified on six benchmark datasets i.e., NUAA Photograph Imposter Database, CASIA, Adience, The Images of Groups Dataset (IOG), The Extended Cohn-Kanade Dataset CK+ and The Japanese Female Facial Expression (JAFFE) Dataset. The proposed model achieved an accuracy of 94.17% for spoofing, 83.26% for age, 95.31% for gender and 96.9% for facial expression. Overall, the modification made in the proposed model has given better results and it will go a long way in the future to support soft-biometric based applications.

## 1. Introduction

**Figure 1:**
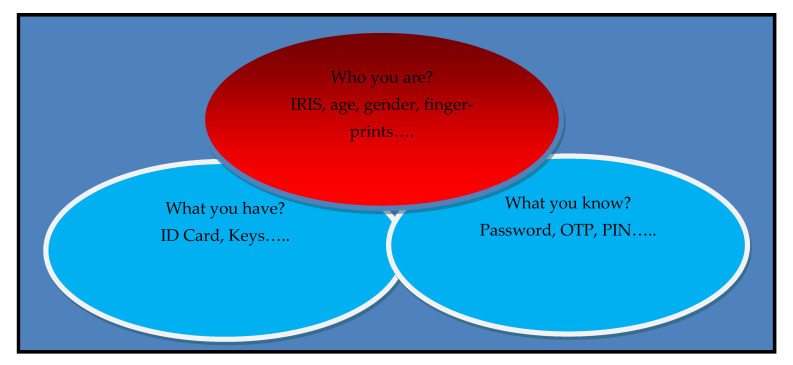
General diagram of Authentication [4].

Today’s modern life is fully automated. Automation is a technique which reduces manual work with the help of information technology. A task which has been done repetitively can be done through automation where human interaction would be less [1]. However, automation comes with security issues. Most industries use a biometric system for the identification of the individual [2,3]. When a person tries to access a particular place or system, then the question which comes into our minds is “Is this the right person to access the particular system or a place?” or “Is this person authorized to access the system or to perform the particular action” or “Does this person belong to a particular organization or state or country?” as shown in Figure 1 [4]. To answer all these questions, we have two methods: the identity of the individual and the ownership of that identity. Both methods could be breached or copied [5,6]. These methods failed because cybercrime is increasing rapidly. According to the reports, many telecommunication and banking sectors are losing a million dollars every year because cyber-attacks on the card and password-based authentication systems. To overcome these security concerns, biometric systems play an important role. The applications where biometric systems play an important role: border control, airport biometrics, financial biometrics, healthcare biometrics, mobile biometrics and many more. In this paper, we are focusing on the healthcare biometric system. Today, the health care industry is trying to adapt the concept of automated authentication of the individual who belongs to the hospital. 

According to the findings of the American Department of Health and Human Services, around 112 million people were affected by health information breaches in February 2016 [5]. In most hospitals, employees can easily access the individual patient’s personal and healthcare-related details. Therefore, there is always a possibility of stealing sensitive information. This information can be used by criminals in different ways such as identity theft, blackmailing individuals and usage of credit/debit card details to commit fraudulent purchases also. In some cases, employees have even stolen the demographic and social security information which could be helpful for them to commit various types of crimes. A biometric system can be categorized into soft biometrics and hard biometrics. Hard biometrics include facial expression, irises and fingerprints, whereas soft biometrics include gender and age information, gait analysis, height, skin color, hair color, etc. Soft computing, based on single biometric information cannot deal with spoofing attacks, large datasets and unacceptable errors, and on the other hand, hard computing alone cannot be used for many applications [7]. Based on that, we proposed a system that includes facial features, gender information and age information and it will also deal with spoofing attacks [8,9,10]. In other words, the proposed system is a combination of soft and hard biometrics which associate a multi-biometric system to enhance the accuracy of the proposed system. The proposed system makes a big difference in the field of the health care system as well as insurance companies. With the help of this system, an authorized person can only access the patient and doctors’ record, which is a big milestone for the health industry [11,12,13].

The proposed system is created primarily for health care personnel, who can use this model to classify, store, share, protect and identify individuals who are member of the healthcare system, as well as cope with record duplication. In the majority of studies, researchers have utilized a single classification approach, such as age, gender, facial expression or face spoofing. In the proposed strategy, we created a hybrid model that employs all four modules for different dataset classifications which distinguishes it from existing methods. There are particular steps that distinguish the proposed technique, and they are as follows:

1. In this paper, we will use 5-layer U-Net-based architecture used for face detection because it gives better segmentation compared to other methods.
2. A spoofing technique will help to identify the unauthorized person, and if a person is authenticated, then only he or she can access the system, which makes our system more secure.
3. Classification will be done through Alex-Net-based architecture, and individuals can be classified according to age, gender and facial expressions. This architecture has the power of giving better accuracy even when the dataset is huge.

The rest of the paper is as follows: In Section 2, we will discuss the related work done by different researchers in the area of age, facial expressions and gender classification using different methods. Section 3 deals with the proposed methodology. Section 4 discusses the results and Section 5 consists of the conclusion and future scope.

## 2. Literature Work

An ample amount of research work has been already done by different researchers in the field of spoofing, age, gender and facial expression. Therefore, it is difficult to discuss all the methods as a single taxonomy; still, we will try to provide a quick and the best overview of the existing approach which is used in the above-mentioned fields. Bruno Peixoto et al. proposed a method that can detect spoofing from low- and high-quality images as well as recaptured and printed images. This method has a good classification rate under different illumination conditions [1]. Yaman AKBULUT et al. had proposed a deep learning-based face spoofing detection method. The proposed method is used for detection of the liveliness of the face from video and liveliness of the face detected using LRF-ELM and CNN methods. The experiments were performed on CASIA and NUAA datasets [4]. Quoc-Tin Phan et al. proposed the LDP-TOP method for the face spoofing detection method. In this method, spatial and temporal information is used for identifying face movements. Experiments were performed on CASIA, ldiap and MSU dataset, methods that are suitable for real-time processing [5]. Graham Desmon Simanjuntak et al. proposed a face spoofing detection method based on color distortion analysis. This method is able to extract color moments and histogram features and PCA is applied for dimensionality reduction and the Naïve bayse classifier is used for classification purposes [6]. Age and gender classification is a prominent area of research because of the increasing number of applications on social media platforms. The performance of the age and gender classification-based systems do not provide significant results as compared to face recognition-based systems. Table 1 provides a little glance at the literature work in this field.

A lot of research is going on in this field for better improvement in terms of accuracy and minimizing the error rate. Gil Levi et al. proposed a CNN-based method for age and gender classification. The CNN model, which was used for implementation, is comparatively shallow, which helps reduce the number of parameters and reduces the chances of over fitting of the data. The size of the training data was comparatively large which would be helpful for improving the accuracy of the model [28]. Eran Eidinger et al. provided a dataset of face images with the label of age and gender. In the proposed method, a support vector machine was used for the estimation of feature attributes which reduced the over-fitting problem also [29]. Afshin Dehghan et al. proposed a fully automated recognition system for face, emotion and gender. The proposed method is implemented using a deep convolutional neural network (DCNN) and a huge amount of dataset was collected which reduced the recognition effort in terms of time [30]. Zakariya Qawaqneh et al. also used DCNN for age estimation. In the proposed method, the system was trained for face recognition and later was used for the estimation of age, which improved the performance of the age estimation model. The proposed method overcame the over-fitting problem, as a model was already trained with a huge number of images and subjects, which leads to better performance of the system [31]. Zukang Liao et al. [32] used a local deep neural network for gender recognition. Instead of using 100 patches per image, only 9 patches per image were extracted, which reduced the training time significantly. The performance of the proposed method was 1% less than the existing age and gender classification methods, and they concluded that mouth and eye regions have more significant features for age classification and gender classification and, therefore, the eye region plays an important role [33]. Salah Eddine-Bekhouche et al. proposed an age and gender classification method using ML-LPQ (Multi-Level Local Phase Quantization) features, and SVM is used to predict age and gender and results are better than existing methods [34]. Ehsan Fazl-Ersi et al. proposed a method for age and gender classification which integrated the various features related to age and gender and it provided more visual information. Only the ROI was extracted from the image, which described the whole image properly. Classification andrecognition were done with the help of a support vector machine and this method improved the classification and recognition accuracy comparatively [35,36]. Modesto Castrillón-Santana et al. used periocular biometrics for gender classification and results were 8% less than the existing state of art method, but this paper suggested the importance of a particular area when the image is not so clear or difficult to capture [37]. The authors had combined the features of a particular face and shoulder area and used a two-stage ensemble classifier for classification purposes. Error rate reduced by 20% compared to other GC-based results [38]. Seyed Mehdi Lajevardi proposed a TPC framework for facial expression recognition which was based on color facial images. These color components provided the extra facial information and the features were extracted using log Gabor filter and the quotient method was applied for the feature selection process [39,40,41]. The classification was done through a multi-class linear discriminant analysis classifier. The proposed method worked and outperformed on low-resolution images and different illumination conditions also. This color information helped in the improvement of the emotion recognition performance of the system [42]. S L Happy et al. proposed a method for facial expression recognition using appearance-based features. Prominent features were extracted from the image which contains most of the facial information. In the proposed methods, a free facial landmark detection technique was used, which gave similar results in terms of performance but significantly better results in terms of execution time, and results were better for low-resolution images also [43]. Jiu-Cheng Xie et al. (2019) used CNN for age classification as age estimation is a multi-class classification problem, and also used the losses function for better optimization of the predicted probability distribution of each class [44,45,46]. The proposed method gave better results on wild face and controlled dataset but the results were not satisfactory in an uncontrolled environment. Jingchun Cheng et al. (2019) proposed a gender classification method based on deep learning techniques in which multiple large face patches were used. Many CNN were trained on each predefined patches which reflected the different image resolution and partial cropping of the image. Further, the decision of every CNN was combined using the voting method which gave the gender classification results accurately [47,48,49]. Every patch was evaluated properly and complementary patches were selected for voting purposes [50,51,52]. The accuracy of the proposed method was comparatively better than the existing techniques [53]. M. Duan et al. (2018) proposed the CNN2ELM method for age estimation which is an aggregation of CNN, ELM. The working of the proposed method is divided into three parts. First, different features were extracted and age grouping had been done through the ELM classification method and the estimation of age had been done through an ELM regressor. The features which were related to age were enhanced by the fusion method. An ELM classifier was used for estimating the accuracy of age groups. The accuracy of the age estimation model was better than state of the art methods [54]. Ke Zhang et al. [55] proposed a CNN and RoR-based age and gender classification approach. To overcome the problem of over-fitting, the RoR model was trained on the dataset. This combination of CNN and RoR had given better results compared to the state of the art methods and CNN-based methods. The accuracy of the system was tested on Adience and the IMDB-WIKI dataset. H. Chen et al. (2019) proposed a face anti-spoofing method based on FARCNN. In this method, a three-way classification approach was developed [56]. A conventional RNN-based method was extended using ROI pooling feature fusion, and crystal loss function was added to the original multi-tasking loss function. To handle the various illumination condition, R-LBP was used. These detectors outperformed and results were better on cross datasets also which shown the effectiveness of the proposed method. W. Sun et al. (2020) proposed a face anti-spoofing method based on FCN-DA-LSA (Fully Convolutional Network with Domain Adaptation and Lossless Size Adaptation). Domain adaption and lossless size adaption improved the accuracy rate of face spoofing detection methods.

Peng Zhihao et al. (2020) [57] proposed a smart home system based on the deep learning concept. In this model, simulation action is involved for lights and blinds to control the lights (ON/OFF). In this model, RNN had taken historical data for simulation and predicted 15 min intervals in a day for lights and blinds. Chia-Shing Tai et al. (2019), [58] proposed a real-time smart home system based on the reinforcement learning concept. This model improved the energy efficiency of smart homes by reducing 53% of the cost, 37.5% peak value and 7.05% PAR value from an average value. Shih-Hsiung Lee et al. (2019) [59] proposed an intelligent home system based on the deep learning concept. In this module, agents recognized the human face as well as pets at home for controlling appliances. The model was trained through VGG-Net architecture using the Image-Net dataset. This model achieved a 96.88% accuracy rate while testing the module. Nada A. Rasheed et al. (2018) [60] proposed texture and color bases classification and reconstruction models for the archaeological fragments with the help of a neural network concept. This module achieved 96.1% accuracy of classification as well as reconstruction of archaeological data. This means that the proposed model achieved a high success rate in the field of reconstruction of archaeological things. Yi-Ping Hung et al. (2018) proposed a simulating- based archaeological excavation in the virtual platform and achieved better results as compared to other state of the art modules [61]. Marco Galli et al. (2018) described the importance of archaeological contexts to reconstruct the original features of archaeological excavation [62]. Athas Agapiou et al. (2018) proposed a Bayesian Neural Network-based archaeological module [63]. This fusion-based module increased the accuracy, recall, precision, classification as well as reconstruction. This fusion-based module achieved a global correlation coefficient of up to 73%. Most of the authors have worked on the accuracy of the face spoofing detection method and most of the researchers had used the traditional method for detection of spoofing attacks and much less research work was done with the help of deep learning techniques and the need to focus on the dimensionality reduction of the feature vector as well accuracy of the secured biometric module. Gender and age classification is an important task in automated face analysis. Most existing approaches for gender classification use only raw/aligned face images after face detection as input. These methods exhibit fair classification ability under constrained conditions, in which face images are acquired under similar illumination with similar poses. The performances of these methods may deteriorate when face images show drastic variances in poses as routinely encountered in real-world data. Nowadays, deep learning-based methods are used for face recognition and results are better than existing methods. The same approach could be applied to age and gender classification, which may improve the accuracy of a classification and will also be helpful for the reduction of processing time in real-time applications.

## 3. Proposed Methodology

**Figure 2:**
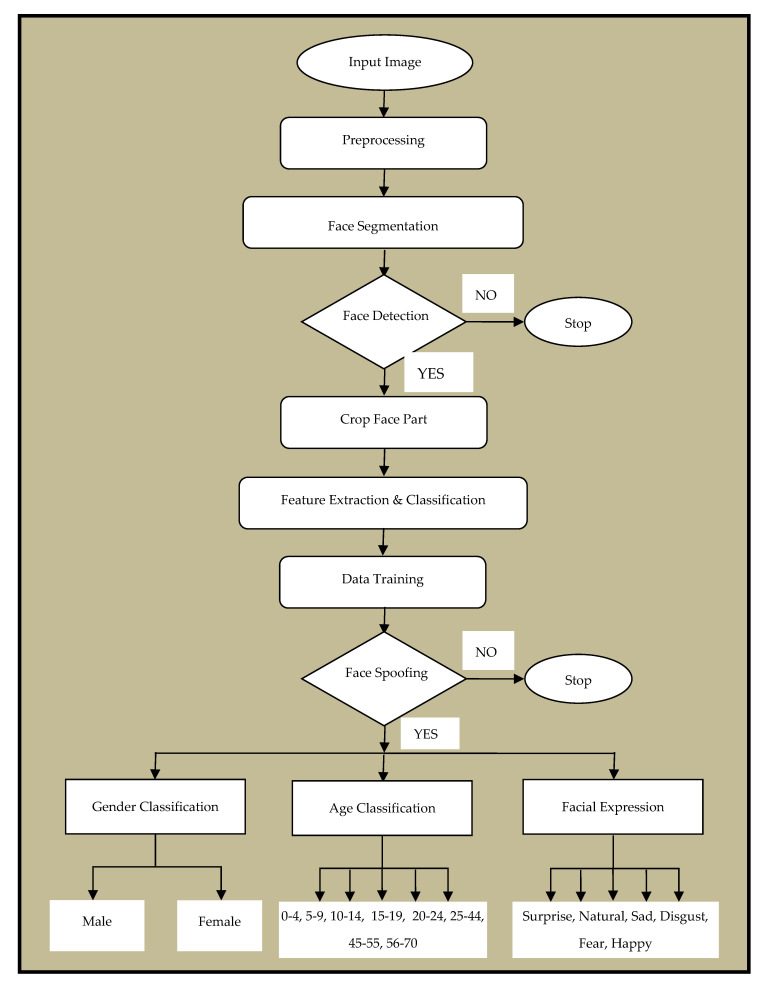
Flow Chart of Proposed Methodology.

A novel hybrid method using deep learning technology is used in this paper to overcome the drawbacks in face spoofing, age and gender recognition. We used U-Net based architecture for Segmentation and Alex-Net for Classification. Until now, as per our knowledge, these architectures have been used for other applications and performed well but have not been used for face spoofing, age and gender recognition. Before using U-Net, we had done a small experiment. Initially, for a particular set of values of hyperparameters for all the architectures i.e., FCN, SegNet and U-Net, training and testing are performed. In this experiment, U-Net architecture performed best among all the three architectures. Therefore, we have selected U-Net architecture for further training and testing. We have trained this architecture from scratch and used all six Benchmark/Standard datasets. We performed our experiment while changing the learning rate on various values, i.e., 10^−2^, 5 × 10^−2^, 10^−3^ and 5 × 10^−4^. Finally, we got a better result when the learning rate is 10^−3^. In the same way, we had done our experiment while changing the batch size of the image i.e., 32, 64, 128 and 264. Finally, we got a better result when the batch size is 128. In Figure 2, the flowchart describes the proposed methodology, in which face detection, spoofing, age and gender with various facial expressions had to be conducted.

The following steps explain the proposed methodology:

1. Image Acquisition:

An image is taken as input which has to convert into a grayscale image. The converted image is then sent to the preprocessing step for image enhancement as well as noise removal. This input image is taken from benchmark datasets used or live through a webcam.

2. Preprocessing:

Preprocessing is performed to remove distortions or other unwanted features while identifying the face part from an image and to extract the proper features of the image corresponding to detect the boundary of the face. In preprocessing, an unwanted feature is removed through the adaptive median filter and the image is resized. In preprocessing, the image is processed through different phases, such as resize, boundary detection and normalization.

**Figure 3:**
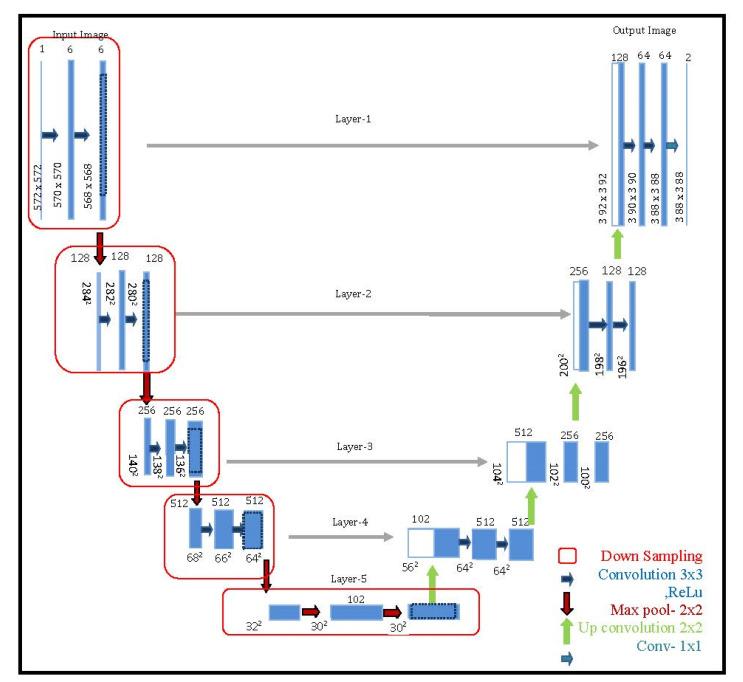
Five-Layer U-Net based Architecture.

3. Segmentation:

After preprocessing, the advanced neural network-based architecture is used for proper segmentation so that the overall face detection rate will increase. The proposed work used 5-layer U- Net-based architecture as shown in Figure 3 and it is one of the fully convolutional neural networks which works with training models of data. Finally, the proposed architecture achieved very powerful segmentation results.

This architecture is parallel in structure and makes very efficient CPUs for image processing applications. It takes less than a second to segment the image. The normal convolution operation is performed using the following steps.

- Define input size of nin×nin×c of 3D volume, where nin is the row and column width, c is channels.
- Define a set of filters, f (kernels, or feature extractors to run on the above matrix), with size 3 or 5.
- The output image of size nout×nout×f 3D volume. (nout is output image width).

U-Net consists of contracting networks with different layers as shown in Table 2, in which all the pooling operations are replaced by upsampling operators, such that the output of the network gives an image of increased resolution, and U-Net performs the classification on every pixel and generates the output with the same size as the input.

An image is read in the form of pixels, having mxnx3 and the convolution layer is formed by sets of filters. These filters are responsible for detecting the specific pattern in the given image. These filters are convolved with the input image resulting in feature maps with different values having the dimension shown in Equation (1).

(N + 2P − F)/S + 1(1)

N = Image Dimension; P = Padding; F = Filter Size; S = Strides

**Figure 4:**
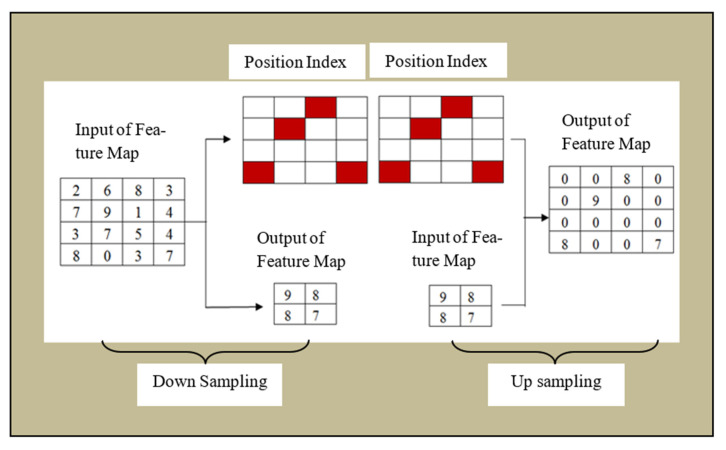
Max-pooling Structure of U-Net/Alex-Net-based Architecture.

Max Pooling was used in the proposed module and it selected the maximum value using filters from a set of pixel values as shown in Figure 4.

The maximum value is extracted at every stride and the remaining are deselected. After max-pooling, the dimension of the matrix is calculated by Equation (2).

(D − F_S_)/S + 1 (2)

D = Dimension of pooling layer; FS = Filter Size; S = Strides

After max-pooling, we applied the activation function. The activation function will perform non-linear transformation over the provided input. Finally, while training and testing, we used the Leaky ReLU function used to overcome the problem of ReLU, and Leaky ReLU function is described in Equation (3):

(3) $$f\left(x\right)=\{\begin{array}{c} 0.01x;for\ldots x<0\\ x;for\ldots x\geq 0 \end{array}$$

Finally, the classification layer performs classification by converting the fully connected output layer to probability for various classes. The classification layer used the SoftMax function to obtain positive values and is described in Equation (4):

(4) $$S\left(yi\right)=\frac{e^{yi}}{\sum_{j=1}^{K}e^{yj}}$$

**Figure 5:**
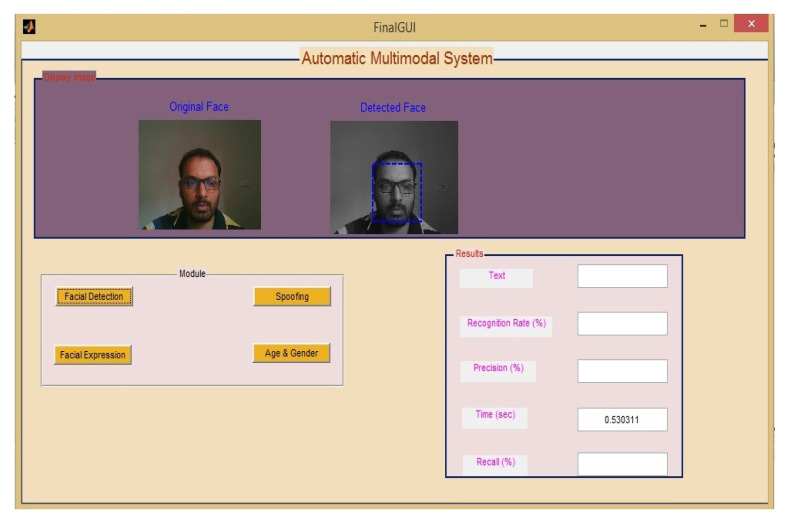
Real-time face detection with the processing time.

Each block in architecture takes an input and passes it through 2 convolution layers of 3 × 3 with a stride of 2 and a max-pooling size of 2 × 2 with a corresponding cropped feature map. Table 2 shows the complete description of U-Net based architecture. For each phase, it takes two convolution layers, and it may have different phases in its architecture. After applying this advanced U-Net based architecture, we achieved an excellent face detection rate on all benchmark datasets as well as in real-time as shown in Figure 5.

**Figure 6:**
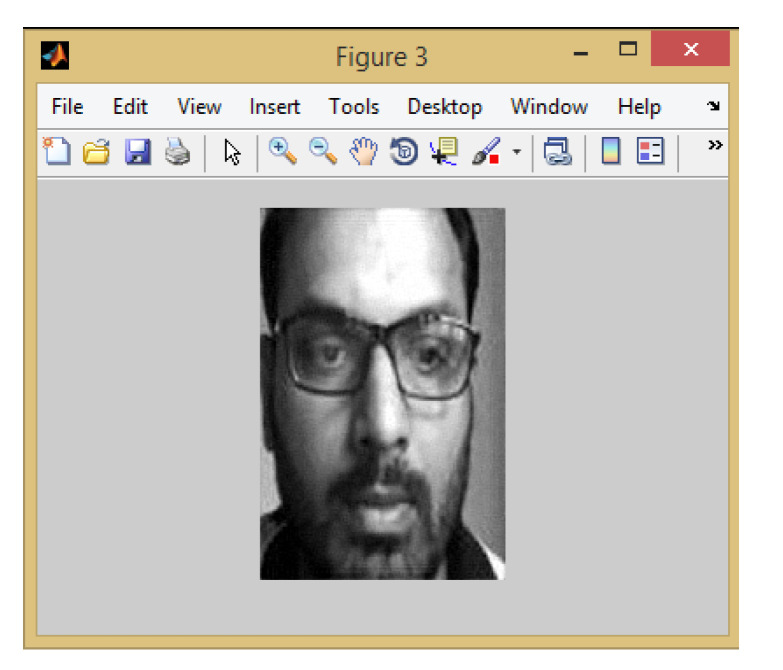
Cropped Image.

When detecting the face in real-time, it takes 0.5 s (less than per second). U-Net based architecture achieved 97% accuracy in real-time and high accuracy on benchmark datasets also. Once the face part is detected from an image, then the face part is cropped for further processing as shown in Figure 6. This means feature extraction.

## 4. Feature Extraction and Classification

**Figure 7:**
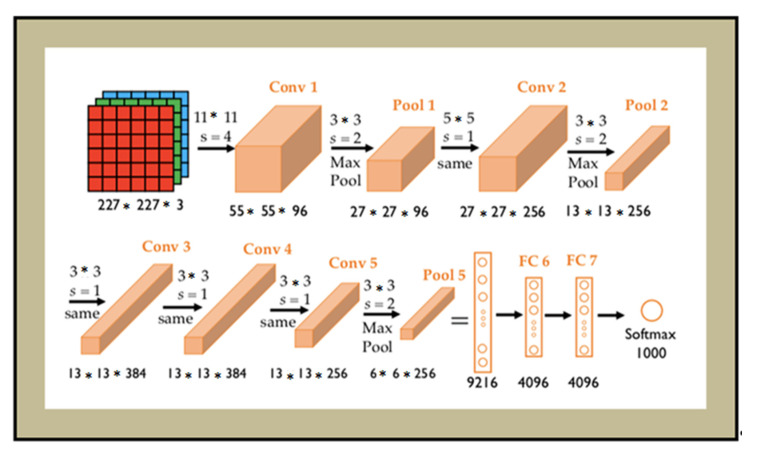
Five-Layer Alex-Net-based Architecture.

After high detection of the face part, feature extraction and classification will play a vital role in the face information of the human being. That is the reason; again, one more advanced neural network (5-Layer Alex-Net architecture) was used for this process.The Alex-Net architecture consists of 5 Convolution layers and 3 fully connected layers. Alex is used to classifying the input of 1000 different classes and generates the output vector of 1000 numbers. Alex uses more than 50 Million parameters and more than 6 lakhs neurons in its implementation. Alex uses ReLu instead of the Tanh function, which makes it six times faster than the existing CNN. The convolution window of Alex is 11 × 11. The window size in the second layer will be reduced to 5 × 5 and followed by 3 × 3 in the consequent layers with max-pooling of 3 × 3 and stride of 2. The layer-wise analysis is discussed in Figure 7.

**Figure 8:**
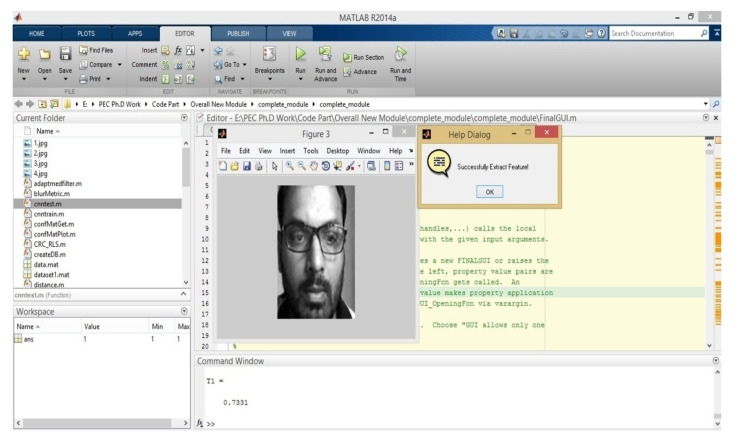
Feature Extraction from cropped Image.

The fifth convolution layer is followed by an overlapping max-pooling layer. This layer outputs into two fully connected layers. The second fully connected layer outputs into the Softmax layer with 1000 class labels. Finally, Figure 8 represents the successful feature extraction from a live image.

After the successful feature extraction process, the last step is classification. This classification process is divided into four sections as shown below:

- Face Spoofing
- Age Classification
- Gender Recognition
- Facial Expression

**Figure 9:**
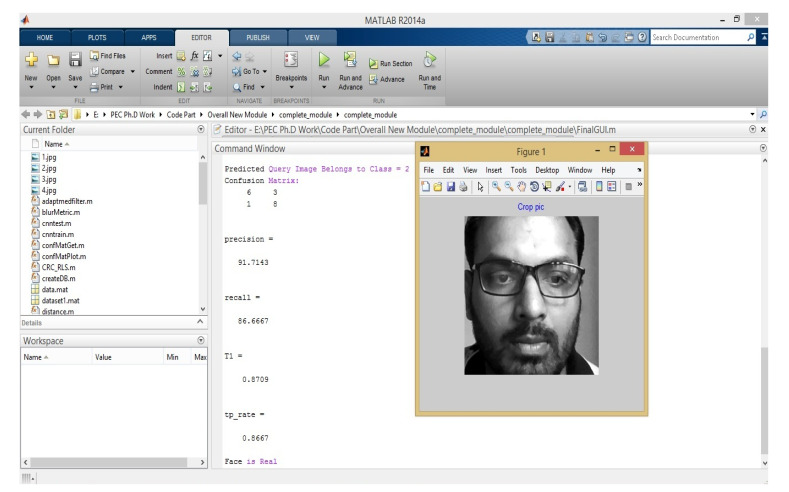
Results of Real-time face detection.

**Figure 10:**
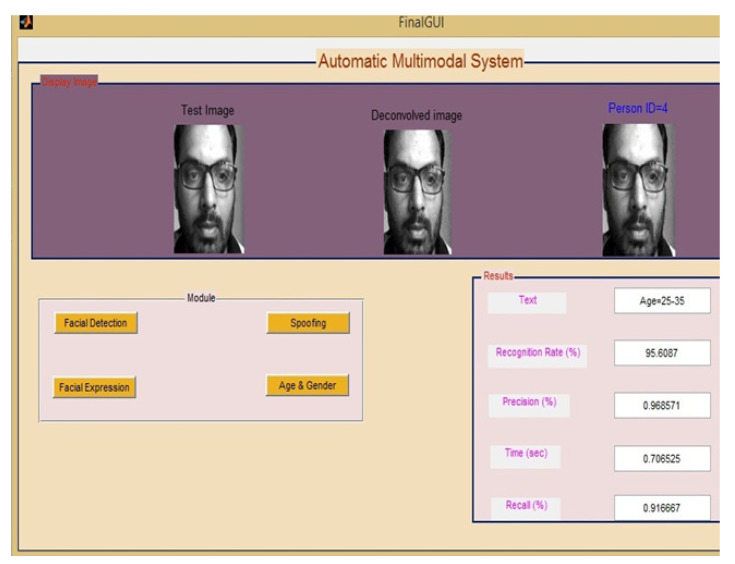
Overall GUI of Proposed Work.

In this step, first of all, Alex-Net architecture will identify that the human face whether the human face is real or fake. If it is fake, then the particular person cannot access the details of the patient or doctors; otherwise, the authorized person can access the entire details of the patient and doctors also. The author himself considers his image as an authorized person and the result of face spoofing is shown in Figure 9 and Figure 10. In the proposed paper, we used four modules with various classifications, i.e., face spoofing (real, fake), gender (male, female), age (0–2, 3–7, 8–13, 14–20, 21–36, 37–60, 60+) and facial expression (anger, disgust, fear, happiness, sadness, surprise).

## 5. Results

The proposed model was developed on Matlab version 2014a and the deep learning toolbox of Matlab was used for the research. For training and testing purpose, the NVIDIA Quadro P4000 graphics card was used as the GPU, which has 8-GB of RAM. For the evaluation process, we have used a 10-fold cross-validation technique to evaluate proposed models by partitioning the benchmark datasets into a training set to train the model, and a test set to evaluate it. During the classification of the human face, Alex-Net architecture is used, which requires values of different hyper-parameters to be set. This is required to obtain the optimum performance of the architecture. These hyper-parameters are epochs, learning rate and dropout and batch size. The optimized values of these hyper parameters for each problem are mentioned in Table 3.

For training of our system, we used six publicly available Benchmark Datasets. We created a combined dataset for each module and then trained our model on these datasets. We converted all the images into the input image size of U-Net architecture, i.e., 572 × 572. For every database, we used 10-fold cross-validation techniques. U-Net-based architecture plays a significant role in pixel-based segmentation and it is more effective with limited datasets images also, which is a common problem for data-scarce computer-vision tasks. Due to the max pool, the resolution of the output will increase. We use binary cross-entropy loss function as our training objective. This architecture worked well during training.

- Early stopping if the validation loss does not improve for 10 continuous epochs.
- Save the weights only if there is an improvement in validation loss.

### 5.1. Dataset Overview

For the sample, the author used his own images for the validation of the proposed work in real-time. For the evaluation of the proposed work, six publicly available benchmark datasets were used. These included NUAA and CASIA for Face Spoofing, Adience and IOG for Age, Adience and IOG for Gender and CK+ and JAFFE [64,65] for Facial Expression for a human face after signing the agreement for their respective organizations. We created a combined dataset for each module, i.e., first module for spoofing (in both the datasets, we have 14K images approximately), the second module for age and gender (in both the datasets, we have 31K approximately) and the last module for facial expression (in both the datasets, we have 11K approximately) and then trained our model on these datasets. The entire description of the benchmark datasets is shown in Table 4.

### 5.2. Evaluation Parameter

The overall proposed work gives better accuracy on all the standard benchmark datasets. Accuracy is the quality or state of being correct or precise. It refers to the closeness of the measured value to the standard value. The mathematic function of the accuracy is shown in Equation (5).

(5) $$Accuracy=\frac{TP+TN}{TP+TN+FP+FN}$$

In Equation (5), TP stands for true positive which indicates the number of positive inputs correctly identified by the model. TN stands for true negative. It is the negative input correctly identified by the model. FP stands for false-positive which means the model identifies the negative input as positive. Finally, FN stands for false-negative and it identifies the positive input as negative.

### 5.3. Evaluation Results in Terms of Accuracy

**Figure 11:**
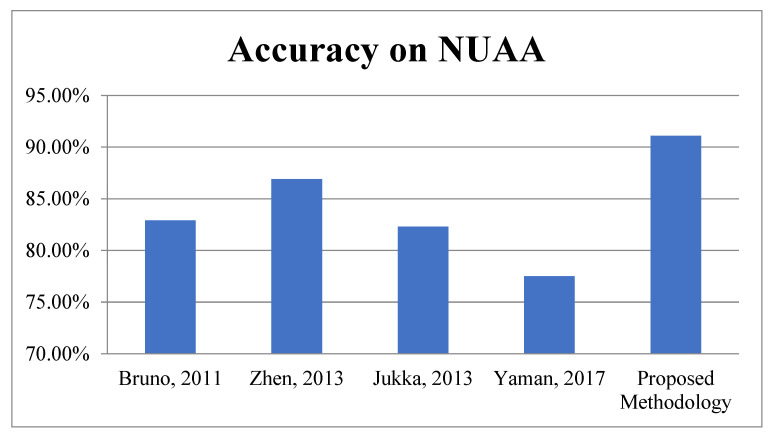
Accuracy Comparison for Face Spoofing on NUAA.

**Figure 12:**
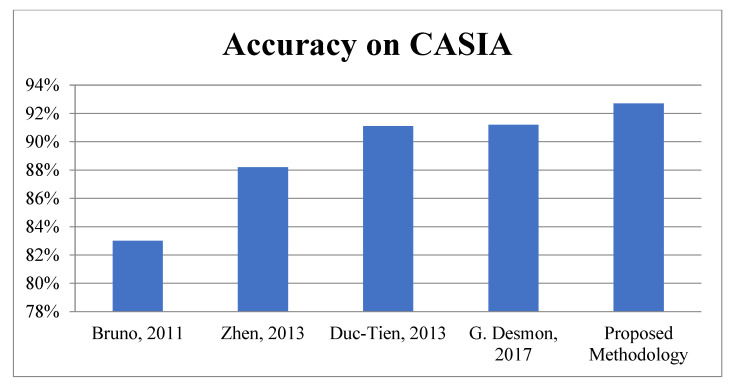
Accuracy Comparison for Face Spoofing on CASIA.

First Module: In the first module, the proposed work is evaluated on benchmark datasets i.e., NUAA and CASIA for face spoofing. According to Table 5, it is very clear the proposed algorithm achieved better accuracy as compared to existing methodologies: 91.1% on NUAA and 92.71% on the CASIA database. The graphical representations of face spoofing results are shown in Figure 11 and Figure 12.

**Figure 13:**
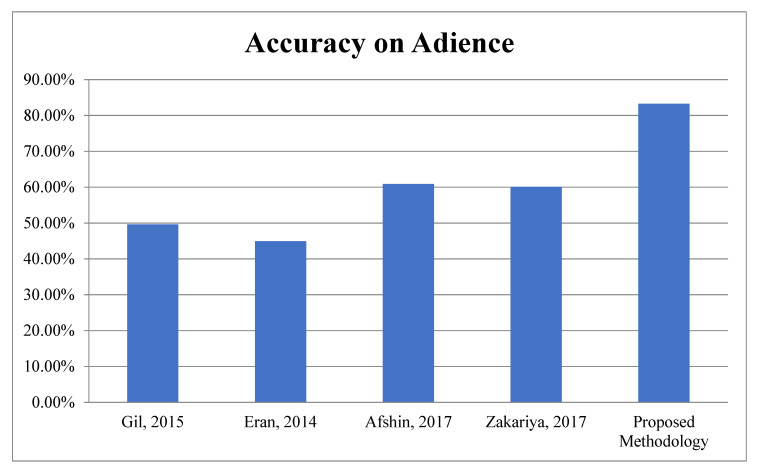
Accuracy Comparison for Age Classification on Adience.

**Figure 14:**
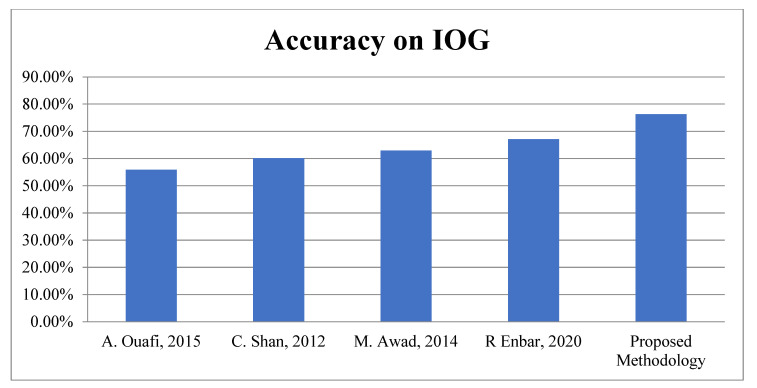
Accuracy Comparison for Age Classification on IOG.

Second Module: In the second module, the proposed work is evaluated on benchmark datasets, i.e., Adience and IOG for Age Classification. According to Table 6, it is very clear the proposed algorithm achieved 83.26 ± 4.3% and 76.3% accuracy for Age and theAdience and IOG database, respectively. The graphical representations of face spoofing results are shown in Figure 13 and Figure 14.

**Figure 15:**
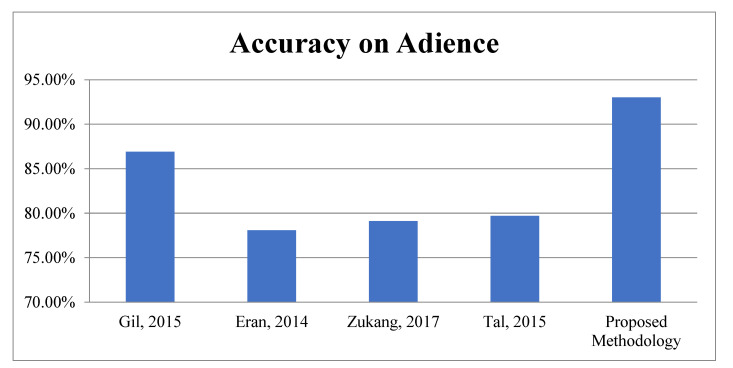
Accuracy Comparison for Gender Classification on Adience.

**Figure 16:**
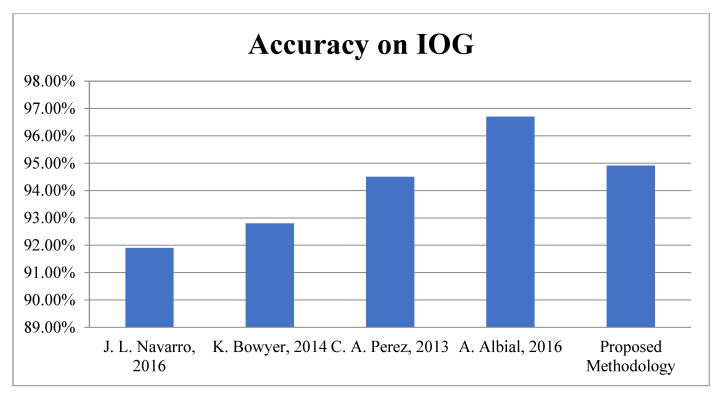
Accuracy Comparison for Gender Classification on IOG.

Third Module: In this module, the proposed work is evaluated on benchmark datasets, i.e., Adience and IOG for gender recognition. According to Table 7, it is very clear the proposed algorithm achieved 93.01 ± 2.03 and 94.91% accuracy for Gender recognition and the Adience and IOG database, respectively. The graphical representations of face spoofing results are shown in Figure 15 and Figure 16.

**Figure 17:**
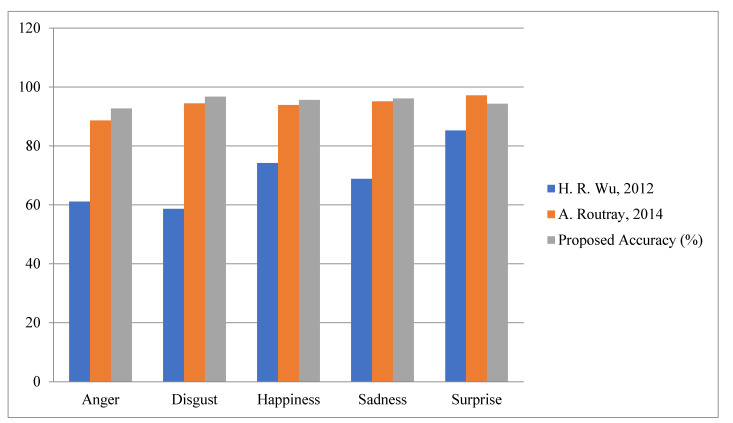
Accuracy Comparison for Facial Expression CK+.

**Figure 18:**
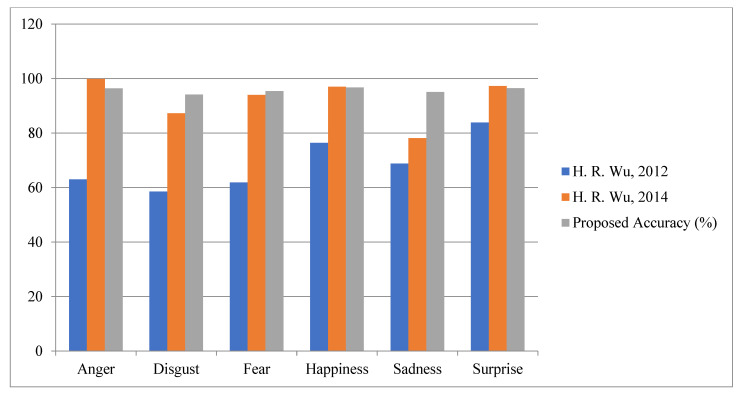
Accuracy Comparison for Facial Expression JAFFE.

Fourth Module: Finally, in the fourth module, the proposed work is evaluated for various facial expressions on benchmark datasets, i.e., CK+ and JAFFE [64,65]. According to Table 8 and Table 9, it is very clear that the proposed algorithm achieved better accuracy for all various expressions, i.e., anger, disgust, happiness, natural, sadness and surprise. The graphical representations of facial expression are shown in Figure 17 and Figure 18.

## 6. Conclusions

In this paper, we have proposed a model of biometric authentication based on face spoofing, facial expression, age, and gender detection which can be used in various places, but is susceptible to intrusion. The significant contribution of this paper is the use of U-Net and AlexNet architecture in collaboration. The U-Net architecture is used for segmentation purposes, and for the classification purpose, Alex-Net is applied. The proposed model is trained and tested on six benchmark datasets i.e., NUAA, CASIA, Adience, IOG, CK+ and JAFFE. Experiments have been performed for spoofing, age and gender classification and facial expression recognition. Comparative analysis has been done with existing methods. Performance of face spoofing is tested on NUAA and CASIA dataset. Accuracy is 91.1% on NUAA and 92.71% on the CASIA dataset, which is better than existing techniques. Accuracy of age classification is tested on Adience and IOG dataset, which is 83.26 ± 4.3% and 76.3%, respectively. Accuracy of gender classification is tested on Adience and IOG dataset, which is 93.01 ± 2.03% and 94.91% respectively. For facial expression recognition, we focused on anger, disgust, happiness, sadness and surprise parameters. The proposed method gives better results for all parameters except surprise. The results prove that our methods have better learning and classification performance compared to existing methods, which can be a boon for maintaining security at vulnerable places by security agencies.

In the future, the model can be trained and tested with real-time datasets and efforts can also be made to make a small prototype of the secured biometric system, which can be useful in various applications i.e., hospitality, military, banking, aviation, the insurance sector, etc. This can also be used in the healthcare sector such as in ICUs and other restricted areas where the respective surgeons/doctors can access the details of patients as well as their reports, especially since day-to-day crime is increasing in the electronic health care system.

## Figures and Tables

**Table 1:** Literature work on existing technology.

Ref.	Author and Year	Dataset	Pre-Processing	Segment-ation	Method	Classification	Performance/Accuracy
[7]	ErnoMakinen, 2008	IIM, FERET	Y	N	Gender	SVM	83.38% accuracy with automatic alignment method.
[8]	Y. Jiang, 2014	Mixed FERET, CAS-PEAL dataset	N	N	Gender	JFLDNNs	Accuracy is 89.63% on mixes dataset, which is good compared to CNN, LBP and CNN + LBP.
[9]	Huajie Chen, 2006	-	N	N	Gender	SVM and AAM	Classifier performance has been improved by adding pseudo examples
[10]	B. Kabasakal, 2018	LFW, IMDB and WIKI	Y	N	Gender	Google Net DNN, SVM	The performance of Google Net is 94.76%, which is better than SVM
[11]	M. Mayo, 2008	---	N	N	Gender	SVMLinear, SVMQuad, RF200	The accuracy of SVMQuad is 92.5%.
[12]	M. Shabanian, 2019	317 MRI data from NDA	Y	N	Age	3DCNN	Achieved sensitivity by 99% and specificity by98.3%.
[13]	M. F. Aydogdu, 2017	MORPH	Y	N	Age	6 layer CNN, ResNet18 and ResNet-34	The performance of ResNet18 and ResNet-34 is better for the age classification problem.
[14]	T. Zheng, 2017	MORPH	Y	N	Age	Deep probabilities CNN	Proposed methods have better accuracy compared to state of theart methods.
[15]	S. Chen, 2017	MORPH album 2	Y	N	Age	SVM multi-class CNN	The proposed method has fewer errors in estimation compared to existing methods
[16]	Fenker S.	Own dataset of 630 images	Y	N	Feature extraction and age prediction	__	69% of accuracy has been achieved.
[17]	Karen Hollingsworth et al., 2009	Own database collected at University	Y	N	Age prediction using IRIS	__	Achieved 70% accuracy
[18]	LVQNet et al., 2011	CASIA-IrisV1	Y	N	Age prediction using IRIS	CNN	LVQNet required 31 iterations for better results
[19]	Salah EddineBekhouche et al., 2015	Groups	N	N	Age and gender classification	SVM with non-linear kernel	Accuracy of age and gender classification is 88.8% 79.1%.
[20]	X. Liu et al., 2017	Adience, CAS-PEAL	N	N	Age and gender classification	Google Net	Accuracy of age and gender classification is about 98%.
[21]	M. R. Dileep et al., 2016	1000 greyscale facial image	N	N	Age and gender classification	FFANN	Accuracy of age and gender classification is about 95%.
[22]	K. Hu et al., 2018	MRI image	N	Y	segmentation	U-Net	The performance of the proposed method is higher compared to other methods
[23]	Sepidehsadat Hosseini et al., 2019	Web face Morph II, FG-Net	N	N	Age and gender, facial expression classification	GF-CapsNet	Performance of Proposed GF- CapsNet is better than plain CNN for age, gender and feature expression recognition.
[24]	Ayesha Gurnani et al.	Adience, AffectNet	N	N	Age and gender, facial expression classification	SAF-BAGE	Performance is better comparatively.
[25]	Zitong Yu et al., 2020	OULU-NPU dataset, CASIA MFSD to Replay-Attack datasets	N	N	Face anti spoofing	CDCN	0.2% ACER in Protocol- 1 of OULU-NPU dataset, HTER from CASIAMFSD to Replay-Attack datasets
[26]	Jiun-Da Lin et al., 2022	Mask Dataset	N	N	Face anti spoofing	ArcFace Classifier (AC)	Performance of the proposed method is better than existing systems.
[27]	Weihua Liu et al., 2021	MIP-2D and MIP-3D, CASIA-SURF	N	Y	Face anti spoofing	D-Net, M-Net	ACER of 0.1071% outperforms all three comparative models with ACER of 0.4152%, 0.3425%, 0.1102%

**Table 2:** Convolution Layers output of U-Net based Architecture.

Phases	Input Image	CNN Layers	Filter	Output Image	Sampling Type	Stride
1	128 × 128 × 3	2	16- 3 × 3	128 × 128 × 16	down	2
2	128 × 128 × 16	2	32- 2 × 2	64 × 64 × 32	down	2
3	64 × 64 × 32	2	2 × 2	32 × 32 × 32	down	2
3	32 × 32 × 32	2	64- 3 × 3	32 × 32 × 64	down	2
4	32 × 32 × 64	2	2 × 2	16 × 16 × 64	down	2
4	16 × 16 × 64	2	128- 3 × 3	16 × 16 × 128	down	2
5	16 × 16 × 128	2	2 × 2	8 × 8 × 128	down	2
5	8 × 8 × 128	2	256- 3 × 3	8 × 8 × 256	up	2
5	8 × 8 × 256	2	-	16 × 16 × 128	up	2
5	16 × 16 × 128	2	-	16 × 16 × 256	up	2
5	16 × 16 × 256	2	128- 3 × 3	16 × 16 × 128	up	2
6	16 × 16 × 128		-	32 × 32 × 64	upsampling to 32 × 32 × 64	2
6	32 × 32 × 64		-	32 × 32 × 128		2
7	32 × 32 × 128	2	64- 3 × 3	32 × 32 × 64		2
7	32 × 32 × 64		-	64 × 64 × 32	upsampling to 64 × 64 × 32	2
	64 × 64 × 32			64 × 64 × 64		2
8	64 × 64 × 64	2	32- 3 × 3	64 × 64 × 32	upsampling to128 × 128 × 16	2
8	128 × 128 × 16		-	128 × 128 × 32		2
9	128 × 128 × 32	2	16- 3 × 3	128 × 128 × 16	128 × 128 × 1	2

**Table 3:** Details of Alex-Net Classifier.

Parameter Name	Spoofing	Age and Gender	Facial Expression
Epochs	35	45	40
Learning Rate	0.001	0.001	0.001
Droupout	0.35	0.40	0.35
Batch Size	128	128	128

**Table 4:** Details of Benchmark/Standard Datasets.

Sr. No.	Dataset Name	Remarks
1	NUAA	Subjects = 15, Real Image = 5000, Fake Image = 7500
2	CASIA	Subjects = 50, Real Image = 500, Fake Image = 450
3	Adience	Total Image = 26,500, 8 Types of Categories
4	IOG	Total Image = 5100, 7 Types of Categories
5	CK+	Subject = 123, Sequence = 593, Total Images = 10,600
6	JAFFE	Total Image: 213, 7 Facial Expression

**Table 5:** Accuracy Comparison for Face Spoofing on NUAA and CASIA.

Sr. No.	References	Accuracy on NUAA	References	Accuracy on CASIA
1	Bruno et al. [1]	82.9%	Bruno et al. [1]	83%
2	Zhen et al. [2]	86.9%	Zhen et al. [2]	88.2%
3	Jukka et al. [3]	82.3%	Duc-Tien et al. [5]	91.1%
4	Yaman et al. [4]	77.51%	G. Desmon et al. [6]	91.2%
5	Proposed Methodology	91.1%	Proposed Methodology	92.71%

**Table 6:** Accuracy Comparison for Age Classification on Adienceand IOG.

Sr. No.	References	Accuracy on Adience	References	Accuracy on IOG
1	Gil et al. [28]	49.6 ± 4.7%	A. Ouafi et al. [34]	55.9%
2	Eran et al. [29]	44.9 ± 2.7%	C. Shan et al. [35]	60.1%
3	Afshin et al. [30]	60.9 ± 4.1%	M. Awad et al. [36]	62.9%
4	Zakariya et al. [31]	60.12%	R Enbar et al. [37]	67.1%
5	Proposed Methodology	83.26 ± 4.3%	Proposed Methodology	76.3%

**Table 7:** Accuracy Comparison for Gender Classification on Adienceand IOG.

Sr. No.	Method	Accuracy on Adience	Method	Accuracy on IOG
1	Gil et al. [28]	86.9 ± 1.6%	J. L. Navarro et al. [38]	91.9%
2	Eran et al. [29]	78.1 ± 1.4%	K. Bowyer et al. [39]	92.8%
3	Zukang et al. [32]	79.13%	C. A. Perez et al. [40]	94.5%
4	Tal et al. [33]	79.7 ± 0.7%	A. Albial et al. [41]	96.7%
5	Proposed Methodology	93.01 ± 2.03%	Proposed Methodology	94.91%

**Table 8:** Accuracy Comparison for Facial Expression CK+.

Expression	H. R. Wu et al. [42]	A. Routray et al. [43]	Proposed Accuracy (%)
Anger	61.09	88.6	92.7
Disgust	58.61	94.41	96.7
Happiness	74.21	93.9	95.6
Sadness	68.81	95.09	96.07
Surprise	85.22	97.17	94.31

**Table 9:** Accuracy Comparison for Facial Expression JAFFE.

Expression	H. R. Wu et al. [42]	H. R. Wu et al. [43]	Proposed Accuracy (%)
Anger	63.01	99.9	96.41
Disgust	58.51	87.3	94.13
Fear	61.9	94.01	95.43
Happiness	76.41	97.01	96.73
Sadness	68.81	78.11	95.07
Surprise	83.91	97.26	96.51

## Data Availability

Not applicable.
