# Correction: Kumar et al. Face Spoofing, Age, Gender and Facial Expression Recognition Using Advance Neural Network Architecture-Based Biometric System. *Sensors* 2022, *22*, 5160

**DOI:** 10.3390/s23218825

**Published:** 2023-10-30

**Authors:** Sandeep Kumar, Shilpa Rani, Arpit Jain, Chaman Verma, Maria Simona Raboaca, Zoltán Illés, Bogdan Constantin Neagu

**Affiliations:** 1Department of Computer Science and Engineering, Koneru Lakshmaiah Education Foundation, Vaddeswaram, Vijaywada 522302, India; 2Department of IT, Neil Gogte Institute of Technology, Kachawanisingaram Village, Hyderabad 500039, India; shilpachoudhary1987@gmail.com; 3College of Computing Sciences and IT, Teerthanker Mahaveer University, Moradabad 244001, India; arpit.computers@tmu.ac.in; 4Department of Media and Educational Informatics, Faculty of Informatics, Eötvös Loránd University, 1053 Budapest, Hungary; chaman@inf.elte.hu (C.V.); illes@inf.elte.hu (Z.I.); 5National Research and Development Institute for Cryogenic and Isotopic Technologies—ICSI Rm, 240050 Ramnicu Valcea, Romania; 6Doctoral School, Polytechnic University of Bucharest, 313 Splaiul Independentei, 060042 Bucharest, Romania; 7Department of Power Engineering, “Gheorghe Asachi” Technical University of Iasi, 700050 Iasi, Romania; bogdan.neagu@tuiasi.ro

## Missing Citation

In the original publication [1], references [64,65] were not cited. The citation has now been inserted in **Section 5. Results**, *Section 5.1. Dataset Overview*, paragraph 1 and should read as follows:

For the sample, the author used his own images for the validation of the proposed work in real-time. For the evaluation of the proposed work, six publicly available benchmark datasets were used. These included NUAA and CASIA for Face Spoofing, Adience and IOG for Age, Adience and IOG for Gender and CK+ and JAFFE [64,65] for Facial Expression for a human face after signing the agreement for their respective organizations. We created a combined dataset for each module, i.e., first module for spoofing (in both the datasets, we have 14K images approximately), the second module for age and gender (in both the datasets, we have 31K approximately) and the last module for facial expression (in both the datasets, we have 11K approximately) and then trained our model on these datasets. The entire description of the benchmark datasets is shown in Table 4.

The citation has also been inserted in **Section 5. Results**, *Section 5.3. Evaluation Results in Terms of Accuracy*, paragraph 4 and should read as follows:

Fourth Module: Finally, in the fourth module, the proposed work is evaluated for various facial expressions on benchmark datasets, i.e., CK+ and JAFFE [64,65]. According to Tables 8 and 9, it is very clear that the proposed algorithm achieved better accuracy for all various expressions, i.e., anger, disgust, happiness, natural, sadness and surprise. The graphical representations of facial expression are shown in Figures 17 and 18.

## References

References 64 and 65 were inserted:

64. Lyons, M.J.; Kamachi, M.; Gyoba, J. Coding Facial Expressions with Gabor Wavelets (IVC Special Issue). *arXiv*
**2020**, arXiv:2009.05938. https://doi.org/10.48550/arXiv.2009.05938.

65. Lyons, M.J. “Excavating AI” Re-excavated: Debunking a Fallacious Account of the JAFFE Dataset. *arXiv*
**2021**, arXiv:2107.13998. https://doi.org/10.48550/arXiv.2107.13998.

The authors state that the scientific conclusions are unaffected. This correction was approved by the Academic Editor. The original publication has also been updated.
